# Association between serum albumin level and incidence of end-stage renal disease in patients with Immunoglobulin A nephropathy: A possible role of albumin as an antioxidant agent

**DOI:** 10.1371/journal.pone.0196655

**Published:** 2018-05-24

**Authors:** Yasuhiro Kawai, Kosuke Masutani, Kumiko Torisu, Ritsuko Katafuchi, Shigeru Tanaka, Akihiro Tsuchimoto, Koji Mitsuiki, Kazuhiko Tsuruya, Takanari Kitazono

**Affiliations:** 1 Department of Medicine and Clinical Science, Graduate School of Medical Sciences, Kyushu University, Fukuoka, Japan; 2 Kidney Unit, National Fukuoka-Higashi Medical Center, Koga, Fukuoka, Japan; 3 Division of Internal Medicine, Fukuoka Dental College, Fukuoka, Japan; 4 Division of Nephrology and Dialysis Center, Japanese Red Cross Fukuoka Hospital, Fukuoka, Japan; 5 Department of Integrated Therapy for Chronic Kidney Disease, Graduate School of Medical Sciences, Kyushu University, Fukuoka, Japan; The University of Tokyo, JAPAN

## Abstract

Serum albumin is the major intravascular antioxidant. Though oxidative stress plays an important role in the pathophysiology of Immunoglobulin A nephropathy (IgAN), the association between serum albumin and the progression of IgAN is not entirely understood. This retrospective cohort study of 1,352 participants with biopsy-proven IgAN determined the associations between serum albumin level and the incidence of end-stage renal disease (ESRD) using a Cox proportional hazards model. Patients were divided into three groups by tertiles of serum albumin level: Low, Middle, and High group (≤3.9 g/dL, 4.0–4.3 g/dL, ≥4.4 g/dL, respectively). During the median 5.1-year follow-up period, 152 patients (11.2%) developed ESRD. Participants in the Low group had a 1.88-fold increased risk for ESRD compared with those in the High group after adjustment for clinical parameters, including urinary protein excretion, and pathological parameters (Oxford classification). We also experimentally proved the antioxidant capacity of albumin on mesangial cells. The intracellular reactive oxygen species and mitochondrial injury, induced by hydrogen peroxide were significantly attenuated in albumin-pretreated mouse mesangial cells and human kidney cells compared with γ-globulin-pretreated cells. Low serum albumin level is an independent risk factor for ESRD in patients with IgAN. The mechanism could be explained by the antioxidant capacity of serum albumin.

## Introduction

Immunoglobulin A nephropathy (IgAN) is the most common type of idiopathic glomerulonephritis throughout the world [1,2]. Previous studies have revealed that hypertension, proteinuria, decreased estimated glomerular filtration rate (eGFR), and pathological severity are the major prognostic factors in patients with IgAN [3–5]. Despite the identification of these risk factors, the long-term renal prognosis of IgAN is still poor [6–8]. It has been reported that the renal survival rate at 20 years was almost 60–70% in IgA nephropathy patients [8,9]. Therefore, to achieve better renal prognosis, further investigation to identify relevant risk factors for renal survival is important in patients with IgAN.

Serum albumin is the most abundant intravascular protein and is widely used for routine clinical examination [10]. It has various physiological functions such as being a key molecule in the regulation of osmotic pressure, a transporter of drugs, fatty acids, and metals [11]. Furthermore, serum albumin is the major intravascular antioxidant, which has more than 70% of free radical-trapping activity in serum [12].

Previous studies have shown that oxidative stress plays an important role in the pathophysiology of IgAN [13–17]. Advanced oxidation protein products (AOPPs), markers of oxidative stress, were higher in the serum of patients with IgAN than those of healthy controls, and levels of AOPPs were correlated with the rate of decline in renal function [16]. Furthermore, we previously reported that low serum bilirubin, which had been recognized as an endogenous antioxidant molecule, was associated with high risk of developing end-stage renal disease (ESRD) in patients with IgAN [18]. From the viewpoint of the antioxidant property of serum albumin, it may act protectively for IgAN. However, few studies have focused on the association between serum albumin and the prognosis of IgAN [3,19]. In this study, we evaluated the association between serum albumin and the incidence of ESRD in IgAN patients by using a large-scale multicenter retrospective cohort. In addition, we performed *in vitro* experiments to investigate whether albumin acted protectively against oxidative stress on mesangial cells, which IgAN primarily affects.

## Materials and methods

### Study design and population

We conducted a multicenter retrospective cohort study of patients with IgAN in Fukuoka, Japan. A total of 1,543 patients who were diagnosed with IgAN by percutaneous kidney biopsy at seven participating institutions (Kyushu University Hospital, Japanese Red Cross Fukuoka Hospital, Hamanomachi Hospital, Munakata Medical Association Hospital, Japan Seamen’s Relief Association Moji Hospital, Karatsu Red Cross Hospital, and Hakujyuji Hospital) between October 1979 and December 2010 were eligible for this study. Patients with Henoch-Schönlein purpura nephritis, lupus nephritis or glomerulonephritis associated liver diseases were not included. Patients with no available clinical data were excluded (n = 191). Finally, 1,352 patients with IgAN were enrolled and followed-up until December 31, 2012.

### Ethical approval

This study was conducted in accordance with the principles of the Declaration of Helsinki and was approved by the Clinical Research Ethics Committee of the Institutional Review Board at Kyushu University Hospital (approval ID 469–06) and all participating institutions. The ethics committee of all participating institutions waived the requirement for written informed consent because of the retrospective nature of the present study.

### Renal outcome

The primary outcome was the incidence of ESRD, which was defined as the initiation of renal replacement therapy (hemodialysis, peritoneal dialysis, kidney transplantation). The secondary outcome was the composite renal outcome defined as the doubling of serum creatinine from baseline or the incidence of ESRD. The outcomes were determined from medical records or by telephone interviews to the clinics and hospitals where the patients visited or to the patients themselves.

### Clinical parameters

The baseline data of the patients were obtained by reviewing medical records at the time of kidney biopsy, including age, sex, body mass index (BMI), systolic blood pressure (SBP), serum albumin, total cholesterol, triglycerides, serum creatinine, and urinary protein excretion defined as 24-h urinary protein excretion or urinary protein-creatinine ratio. Serum albumin was measured by the colorimetric method. Total cholesterol and triglycerides were measured by the enzymatic method. Serum creatinine was measured by Jaffe’s method until April 1988 and by the enzymatic method from May 1988 at Kyushu University. At the other institutions, serum creatinine was measured by Jaffe’s method until December 2000 and by the enzymatic method from January 2001. The serum creatinine levels of Jaffe’s method were converted to the enzymatic method levels by subtracting 0.207 mg/dL according to a previous study [20]. Urinary protein was measured by the colorimetric method, and urinary creatinine was measured by the enzymatic method. The eGFR in patients younger than 18 years old was calculated using the Schwartz formula [21,22]. The eGFR in patients 18 years or older was calculated by the Japanese guideline equation: eGFR (mL/min/1.73 m^2^) = 194 × Cr^−1.094^ × age^−0.287^ (if female, × 0.739) [23].

### Pathological parameters

The original kidney biopsy specimens were reviewed by pathologists and were evaluated according to the definition of the Oxford classification [24]. The mesangial hypercellularity score (M) was defined as M0 if the score was ≤0.5 and M1 if the score was >0.5. Endocapillary hypercellularity (E) and segmental glomerulosclerosis (S) were defined as E0 and S0 if absent and as E1 and S1 if present respectively. S score included both segmental glomerulosclerosis and tuft adhesions. Tubular atrophy/interstitial fibrosis (T) was semi-quantitatively classified according to the percentage of lesions in the cortical area (T0 for 0–25%, T1 for 26–50%, and T2 for >50%). In addition, we also categorized extracapillary proliferation (cellular or fibrocellular crescents) as C0 if absent and as C1–2 if present according to the Oxford report [25].

### *In vitro* experiments

#### Cell culture

The mouse glomerular mesangial cells, SV40 MES-13 cell line (American Type Culture Collection, Manassas, VA) and human embryonic kidney (HEK) 293 FT cells (Thermo Fisher Scientific, Waltham, MA) were used. Both these cells were cultured in Dulbecco’s modified Eagle’s medium (DMEM; Nacalai Tesque, Kyoto, Japan) supplemented with 10% fetal bovine serum (Sigma-Aldrich, St. Louis, MO) at 37°C in an atmosphere of 5% CO_2_. Before exposure to hydrogen peroxide, cells were serum starved for several hours in serum-free DMEM after growing to 60–70% confluence as needed.

#### Exposure of cells to hydrogen peroxide

Cells were incubated with various concentration of bovine serum albumin (BSA; A-9576, Sigma-Aldrich), γ-globulin (Wako Pure Chemical Industries, Tokyo, Japan), or the same amount of phosphate buffered saline (PBS) in serum-free DMEM for one hour before exposure to hydrogen peroxide at 37°C. For HEK 293 cells, human serum albumin (HSA; A9731, Sigma-Aldrich) was used instead of BSA. Cells were then exposed to 500 μM hydrogen peroxide (Wako Pure Chemical Industries) for six hours.

#### Detection of intracellular ROS

Hydrogen peroxide-induced oxidative stress was assessed using dihydroethidium (DHE, Sigma-Aldrich) and CellROX green reagent (Life Technologies, Grand Island, NY). After exposure to 500 μM hydrogen peroxide for 6 h, 10 μM DHE or 5 μM CellROX green reagent was added to each cell on 4 chamber slides (Thermo Fisher Scientific) and incubated with DHE for 15minutes or with CellROX green reagent for 30 min at 37°C in DMEM. Cells were then fixed in 4% paraformaldehyde and mounted with VECTASHIELD HardSet Antifade Mounting Medium with DAPI (Vector Laboratories, Burlingame, CA). Images were obtained using a confocal laser scanning microscopy (LSM 710; Carl Zeiss, Oberkochen, Germany). Fluorescence intensities were measured using ImageJ software (http://rsb.info.nih.gov/ij/). For superoxide quantification, cells were treated with DHE dye as above and 1.0 μg/mL Hoechst 33342 (Wako Pure Chemical Industries) in black walled clear bottom 96 well plates (Corning Life Sciences, Tewksbury, MA) for 15 min and were scanned in a fluorescent plate reader (PerkinElmer Ensight: Perkin-Elmer Inc., Wellesley, MA) at λ_ex_ = 535 nm, λ_em_ = 610 nm for DHE, and λ_ex_ = 350 nm, λ_em_ = 460 nm for Hoechst 33342. The DHE fluorescence intensity per cell was determined by dividing DHE fluorescence by the Hoechst 33342 fluorescence.

#### Detection of mitochondrial damage

Hydrogen peroxide-induced mitochondrial damage was assessed using MitoTracker Red CMXRos (Molecular Probes, Eugene, OR). After exposure to hydrogen peroxide, 100 μM MitoTracker Red CMXRos, which accumulates in mitochondria by membrane potential, was added to cells and incubated at 37°C for 30 min. Cells were fixed in 4% paraformaldehyde and mounted with VECTASHIELD HardSet Antifade Mounting Medium with DAPI. Images were obtained using a confocal laser scanning microscopy, and fluorescence intensities were measured using ImageJ software.

### Statistical analysis

The patients were divided into three groups according to tertiles of serum albumin levels: Low group (serum albumin ≤3.9 g/dL), Middle group (serum albumin 4.0–4.3 g/dL), and High group (serum albumin ≥4.4 g/dL). We determined the albumin tertiles as values that divided the total number of patients by three. The baseline data were presented as percentages for categorical variables and median (interquartile range) for continuous variables. The linear trends of continuous variables and categorical variables of baseline data across tertiles of serum albumin were evaluated using a linear regression model and a Cochran-Armitage trend test. The renal survival rate according to the three groups was depicted by the Kaplan-Meier method and compared by the log-rank test. The age- and sex-adjusted and multivariable-adjusted hazard ratios (HRs) with 95% confidence intervals (CIs) of the renal survival according to the three groups were calculated using a Cox proportional hazards model. The assumption of the proportional hazards was examined graphically using the log cumulative hazard plots for the outcomes according to the three groups. The multivariable-adjusted model was adjusted for age, sex, BMI, SBP, eGFR, urinary protein, total cholesterol, and triglycerides (model 1), and adjusted for model 1 parameters plus the pathological parameters of the Oxford classification (M, E, S, T, and C) (model 2). The effect of heterogeneity on serum albumin levels between subgroups was evaluated by adding an interaction term to the relevant multivariable Cox proportional hazard model. The differences between groups in the *in vitro* studies were compared by one-way analysis of variance (ANOVA) followed by Tukey-Kramer post hoc analysis. A two-tailed *P*-value <0.05 was considered statistically significant for all analyses excluding tests of interaction, in which a *P*-value <0.10 was considered significant. The JMP version 11 for Windows (SAS Institute, Cary, NC) was used for statistical analysis.

## Results

### Distribution of serum albumin levels

**Fig 1:**
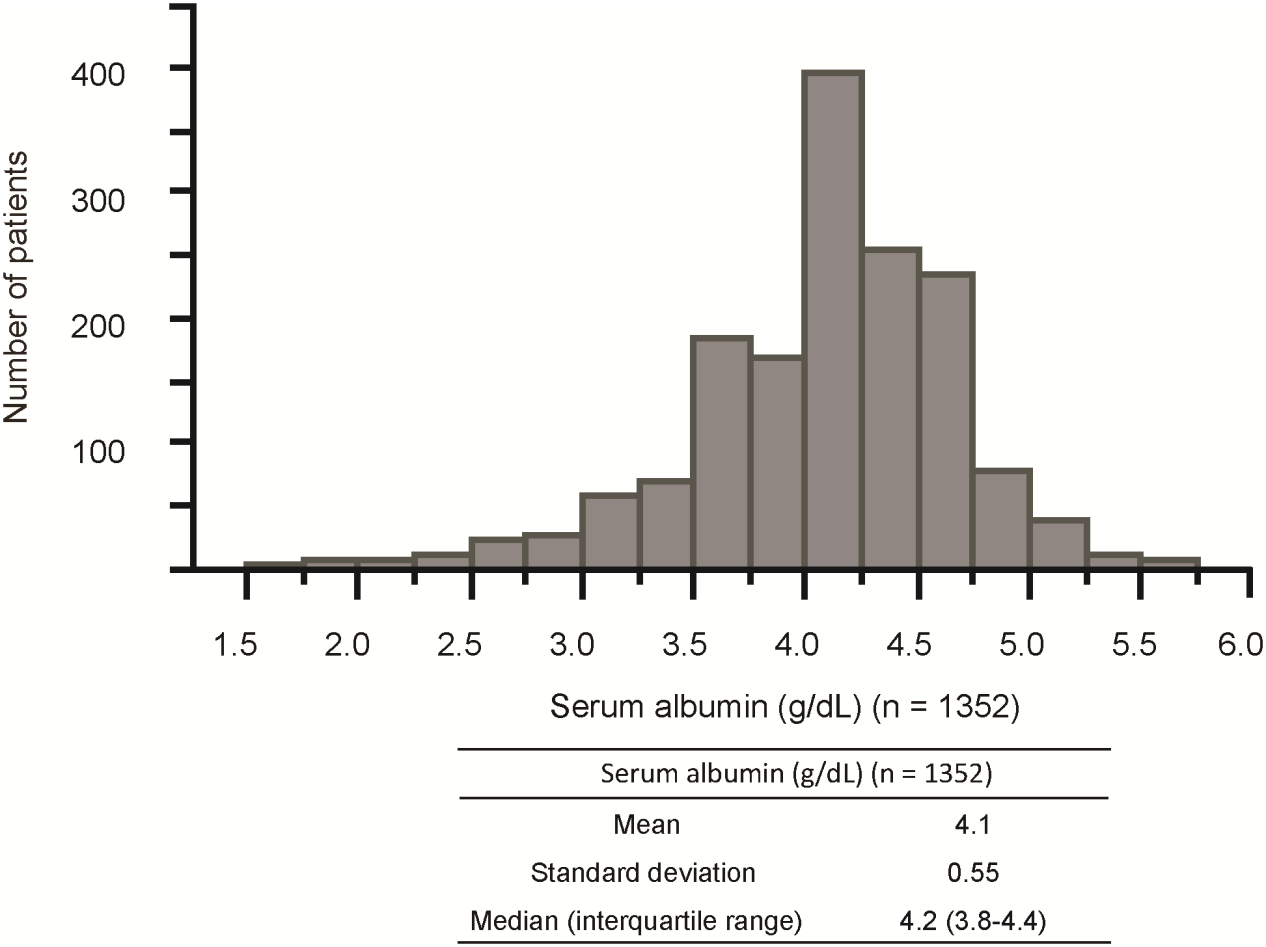
Distribution of baseline serum albumin levels. Histogram of serum albumin levels in the study population (n = 1,352).

Fig 1 shows the histogram of serum albumin levels in the study population (n = 1,352) at baseline. Serum albumin levels showed normal distribution. The mean and the standard deviation were 4.1 g/dL and 0.55 g/dL, respectively. The median and the interquartile range were also 4.2 g/dL and 3.8–4.4 g/dL, respectively.

### Baseline characteristics according to tertiles of serum albumin

The clinical and pathological features of the study population (n = 1,352) based on tertiles of serum albumin at baseline are summarized in Table 1. Patients with lower serum albumin levels were significantly older and had higher SBP, urinary protein excretion, total cholesterol, and triglycerides. In contrast, they were less frequently male and had lower eGFR. In pathological parameters, the population of M1, E1, S1, T1 or T2, and C1–2 was significantly higher in patients with lower serum albumin levels.

**Table 1:** Baseline characteristics according to tertiles of serum albumin levels.

Variable	Unit	Total (n = 1,352)	Serum albumin levels (g/dL)			*P* for trend
			Low group ≤3.9 (n = 458)	Middle group 4.0–4.3 (n = 456)	High group ≥4.4 (n = 438)	
**Clinical parameters**						
**Age**	years	35 (23–47)	42 (27–53)	31 (23–45)	26 (20–39)	<0.001
**Sex (male)**	%	45.3	39.7	45.0	51.4	<0.001
**BMI**	kg/m^2^	22 (20–24)	22 (20–24)	22 (20–24)	21 (20–24)	0.06
**SBP**	mmHg	122 (113–138)	128 (114–144)	122 (114–134)	120 (112–132)	<0.001
**eGFR**	mL/min/1.73 m^2^	78 (58–99)	62 (45–82)	82 (63–101)	89 (71–104)	<0.001
**Urinary protein excretion**	g/24 h	0.79 (0.27–1.75)	1.67 (0.79–3.19)	0.59 (0.24–1.25)	0.40 (0.14–0.9)	<0.001
**Total cholesterol**	mg/dL	196 (169–227)	213 (183–249)	190 (164–217)	188 (166–216)	<0.001
**Triglycerides**	mg/dL	102 (70–151)	117 (83–171)	93 (66–145)	93 (61–142)	<0.001
**Pathological parameters**						
**M1**	%	12.4	19.7	11.8	5.3	<0.001
**E1**	%	39.9	54.6	63.2	37.2	0.01
**S1**	%	76.4	83.2	74.3	71.5	<0.001
**T0**	%	74.2	65.5	80.0	77.2	<0.001
**T1**	%	15.8	20.3	14.0	13.0	
**T2**	%	10.0	14.2	5.9	9.8	
**C1–2**	%	34.7	39.1	32.7	32.2	0.03

Continuous variables are presented as median (interquartile range) and categorical variables as percentages.

Abbreviations: BMI, body mass index; C, crescent score; E, endocapillary hypercellularity score; eGFR, estimated glomerular filtration rate; M, mesangial hypercellularity score; S, segmental glomerulosclerosis score; SBP, systolic blood pressure; T, tubular atrophy/interstitial fibrosis score.

### Association between serum albumin and the incidence of ESRD or composite renal outcome

**Fig 2:**
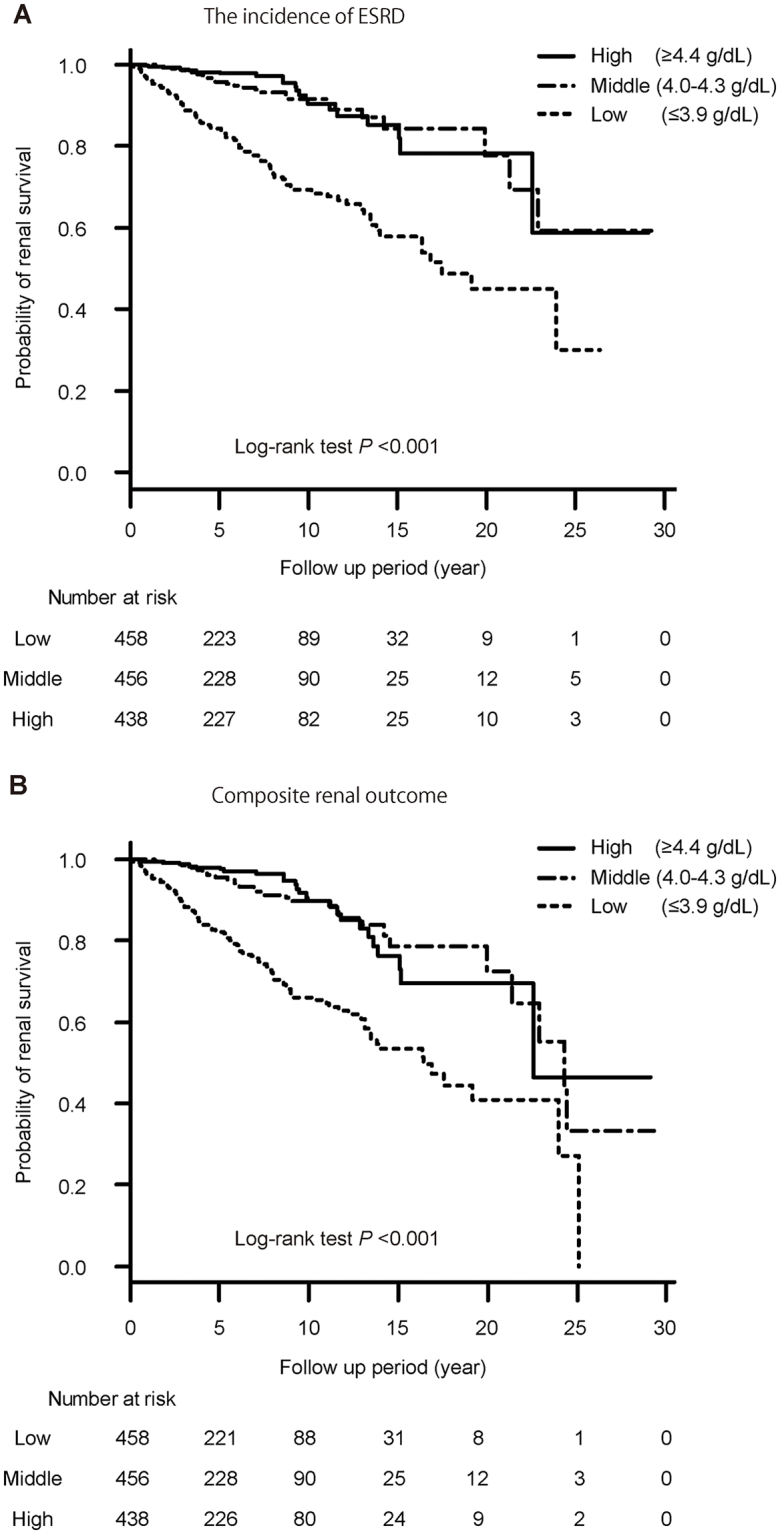
The probability of renal survival according to serum albumin levels. Renal survival according to serum albumin levels during the follow-up period in the study population (n = 1,352).

During the median 5.1-year follow-up period, 152 patients (11.2%) developed ESRD and 178 (13.2%) patients had a composite renal outcome. Fig 2 shows the unadjusted probability of renal survival according to the three groups (Fig 2A: for the incidence of ESRD, Fig 2B: for the composite renal outcome). The probability of renal survival decreased with lower serum albumin (*P* <0.001). Patients in the Low group had a 4.86-fold (95% CI, 3.06–8.07), 2.09-fold (95% CI, 1.28–3.54), and 1.88-fold (95% CI, 1.15–3.20) higher risk of the incidence of ESRD compared to the High group, after age- and sex-adjustment, and after adjustment for clinical parameters (model 1), and for clinical and pathological parameters (model 2), respectively (Table 2). Furthermore, every 1.0-g/dL decrease in serum albumin levels was associated with a 1.89-fold (95% CI, 1.37–2.60) increased risk of the incidence of ESRD after adjusting for the above-mentioned confounding factors. Patients in the Low group also had a higher risk of composite renal outcome compared with those in the High group (Table 3). In addition, every 1.0-g/dL decrease in serum albumin levels was also associated with a 2.06-fold (95% CI, 1.09–3.99) increased risk of the incidence of ESRD (S1 Table) even when we added C-reactive protein to confounding factors in order to exclude the effect of systemic inflammation.

**Table 2:** Age- and sex-adjusted or multivariable-adjusted hazard ratios for the incidence of ESRD.

Variable	No. of events	No. of patients	Age- and sex-adjusted			Multivariable-adjusted (model 1)^a^			Multivariable-adjusted (model 2)^b^		
			HR (95% CI)	*P* value	*P* for trend	HR (95% CI)	*P* value	*P* for trend	HR (95% CI)	*P* value	*P* for trend
**Serum albumin levels (g/dL)**											
**High group (≥4.4)**	21	438	1.00 (reference)		<0.001	1.00 (reference)		<0.001	1.00 (reference)		0.003
**Middle group (4.0–4.3)**	27	456	1.26 (0.71–2.26)	0.43		1.01 (0.57–1.82)	0.97		0.97 (0.54–1.75)	0.92	
**Low group (≤3.9)**	104	458	4.86 (3.06–8.07)	<0.001		2.09 (1.28–3.54)	0.003		1.88 (1.15–3.20)	0.01	
**Every 1.0-g/dL decrease in albumin**	152	1,352	4.26 (3.25–5.55)	<0.001		2.02 (1.47–2.75)	<0.001		1.89 (1.37–2.60)	<0.001	

^a^Model 1: Adjusted for age, sex, SBP, urinary protein excretion, BMI, eGFR, total cholesterol, and triglycerides.

^b^Model 2: Model 1 plus M, E, S, T, and C.

Abbreviations: BMI, body mass index; C, crescent score; CI, confidence interval; eGFR, E, endocapillary hypercellularity score; estimated glomerular filtration rate; ESRD, end-stage renal disease; HR, hazard ratio; M, mesangial hypercellularity score; S, segmental glomerulosclerosis score; SBP, systolic blood pressure; T, tubular atrophy/interstitial fibrosis score.

**Table 3:** Age- and sex-adjusted or multivariable-adjusted hazard ratios for the doubling of serum creatinine from baseline or the incidence of ESRD.

Variable	No. of events	No. of patients	Age- and sex-adjusted			Multivariable-adjusted (model 1)^a^			Multivariable-adjusted (model 2)^b^		
			HR (95% CI)	*P* value	*P* for trend	HR (95% CI)	*P* value	*P* for trend	HR (95% CI)	*P* value	*P* for trend
**Serum albumin levels (g/dL)**											
**High group (≥4.4)**	27	438	1.00 (reference)		<0.001	1.00 (reference)		<0.001	1.00 (reference)		0.003
**Middle group (4.0–4.3)**	35	456	1.17 (0.71–1.96)	0.53		1.02 (0.61–1.70)	0.95		0.98 (0.59–1.65)	0.94	
**Low group (≤3.9)**	116	458	3.89 (2.56–6.11)	<0.001		2.03 (1.31–3.25)	0.001		1.85 (1.19–2.97)	0.006	
**Every 1.0-g/dL decrease in albumin**	178	1,352	3.90 (3.02–5.02)	<0.001		2.14 (1.60–2.87)	<0.001		2.02 (1.50–2.72)	<0.001	

^a^Model 1: Adjusted for age, sex, SBP, urinary protein excretion, BMI, eGFR, total cholesterol, and triglycerides.

^b^Model 2: Model 1 plus M, E, S, T, and C.

Abbreviations: BMI, body mass index; C, crescent score; CI, confidence interval; E, endocapillary hypercellularity score; eGFR, estimated glomerular filtration rate; ESRD, end-stage renal disease; HR, hazard ratio; M, mesangial hypercellularity score; S, segmental glomerulosclerosis score; SBP, systolic blood pressure; T, tubular atrophy/interstitial fibrosis score

### Association between serum albumin and incidence of ESRD in stratified subgroups according to baseline characteristics

**Fig 3:**
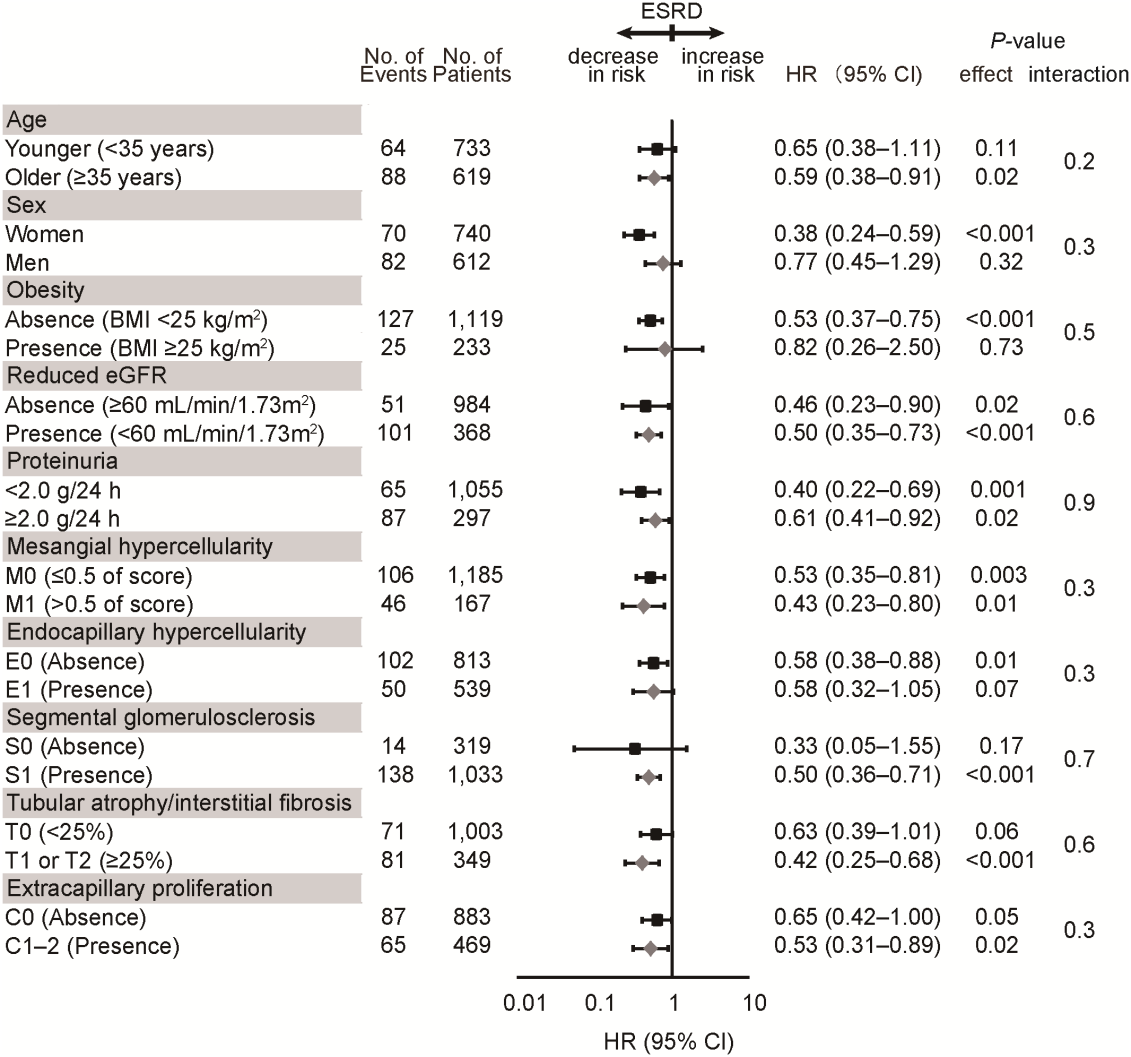
Multivariable-adjusted hazard ratios for the incidence of end-stage renal disease in subgroups stratified according to baseline characteristics. Multivariable-adjusted HRs and 95% CIs for the incidence of end-stage renal disease (ESRD) for every 1.0-g/dL increase in serum albumin levels in various subgroups stratified according to baseline characteristics. The results were adjusted for age, sex, SBP, urinary protein excretion, BMI, eGFR, total cholesterol, triglycerides, M, E, S, T, and C. Variables relevant to the subgroups were excluded from each model. Abbreviations: BMI, body mass index; C, crescent score; CI, confidence interval; E, endocapillary hypercellularity score; eGFR, estimated glomerular filtration rate; HR, hazard ratio; M, mesangial hypercellularity score; S, segmental glomerulosclerosis score; SBP, systolic blood pressure; T, tubular atrophy/interstitial fibrosis score.

To evaluate the consistency of the association between serum albumin and the incidence of ESRD, we performed subgroup analysis stratified by potential confounders (Fig 3). In subgroup analysis, no significant interactions were obtained between serum albumin and baseline characteristics, such as age, sex, obesity (BMI ≥25 kg/m^2^), reduced eGFR (<60 mL/min/1.73 m^2^), proteinuria (urinary protein excretion ≥2.0 g/24 h), the pathological parameters of the Oxford classification (M, E, S, T, and C scores).

### Effect of albumin on the decrease in the intracellular reactive oxygen species (ROS) and mitochondrial damage induced by oxidative stress in mouse mesangial cells and human kidney cells

**Fig 4:**
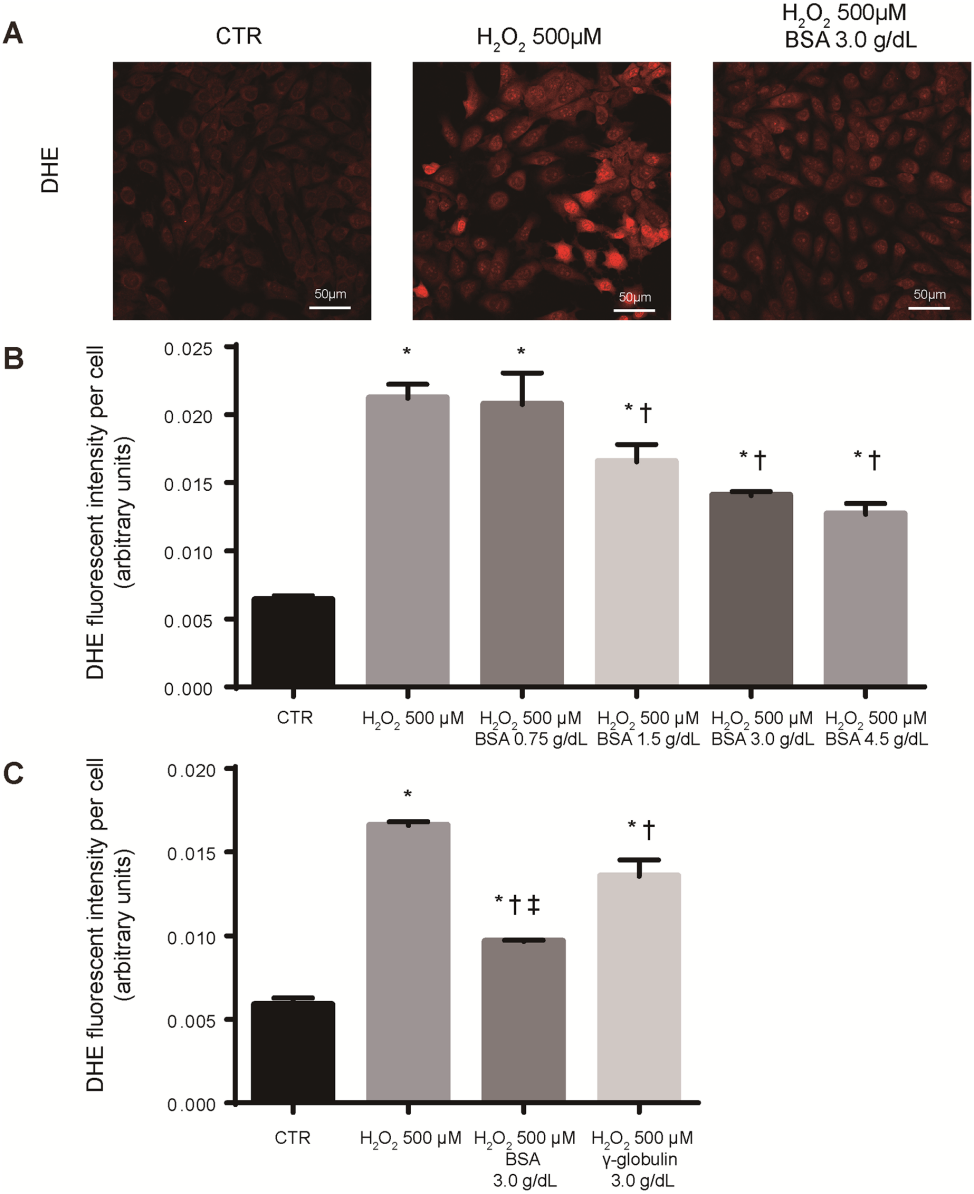
Albumin led to the reduction of intracellular superoxide anion level induced by hydrogen peroxide in mouse mesangial cells. (A) Representative image of intracellular superoxide anion detected by dihydroethidium (DHE) staining in SV40 MES-13 cells after 6-h exposure to hydrogen peroxide (500 μM) with or without bovine serum albumin (BSA, 3.0 g/dL). As a control (CTR), we used the cells that were not exposed to hydrogen peroxide. (B, C) The DHE fluorescent intensity per cell of SV40 MES-13 cells that were exposed to hydrogen peroxide with or without BSA or with γ-globulin. MES-13 cells were exposed to 500 μM hydrogen peroxide for 6 h with or without BSA (0.75, 1.5, 3.0, and 4.5 g/dL) or with 3.0 g/dL γ-globulin, and the DHE fluorescent intensity per cell was determined by the fluorescent plate reader. As a CTR, we used the cells that were not exposed to hydrogen peroxide. Data are presented as the mean ± standard error of three independent experiments, each of which was repeated at least three times with similar results obtained. * *P* <0.05 vs. CTR, † *P* <0.05 vs. H_2_O_2_ 500 μM, ‡ *P* <0.05 vs. H_2_O_2_ 500 μM + γ-globulin.

In our study, albumin at the time of renal biopsy was associated with the prognosis of IgAN. Considering the antioxidant activity of albumin, we hypothesized that albumin has not only a role as a marker but also a direct renal protective effect. To confirm this hypothesis, we investigated in *in vitro* experiments whether albumin was protective against intracellular damage caused by oxidative stress in kidney cells. We used mesangial cells as representative cells of the kidney. Mesangial cells are predominantly injured in patients with IgAN. Mesangial cells are isolated from a blood component with fenestrated vascular endothelial cells, and there is no basal membrane between the mesangial cell and glomerular capillary [26,27]. Therefore, mesangial cells easily contact with serum proteins. From this point, we hypothesized that antioxidant property of serum albumin might act protective effect not only intravascular but also in mesangial cells. To test this hypothesis, we analyzed intracellular superoxide anion, which was a major ROS, in mouse mesangial cells following hydrogen peroxide with and without BSA incubation by DHE. Using confocal fluorescent microscopy, we observed increased intracellular superoxide anion after 500 μM hydrogen peroxide stimulation and a decrease when cells were pretreated with 3.0 g/dL BSA (Fig 4A). Consistent with results of the microscopic examination, the intensity of DHE fluorescent per cell induced by 500 μM hydrogen peroxide decreased in BSA-pretreated cells in a dose-dependent manner (Fig 4B). Furthermore, DHE fluorescent intensity per cell induced by 500 μM hydrogen peroxide was significantly lower in 3.0 g/dL BSA-pretreated group than in the same amount of γ-globulin-pretreated group, the second most abundant protein in the serum, although a decrease in DHE fluorescent intensity was also observed in γ-globulin-pretreated group (Fig 4C).

**Fig 5:**
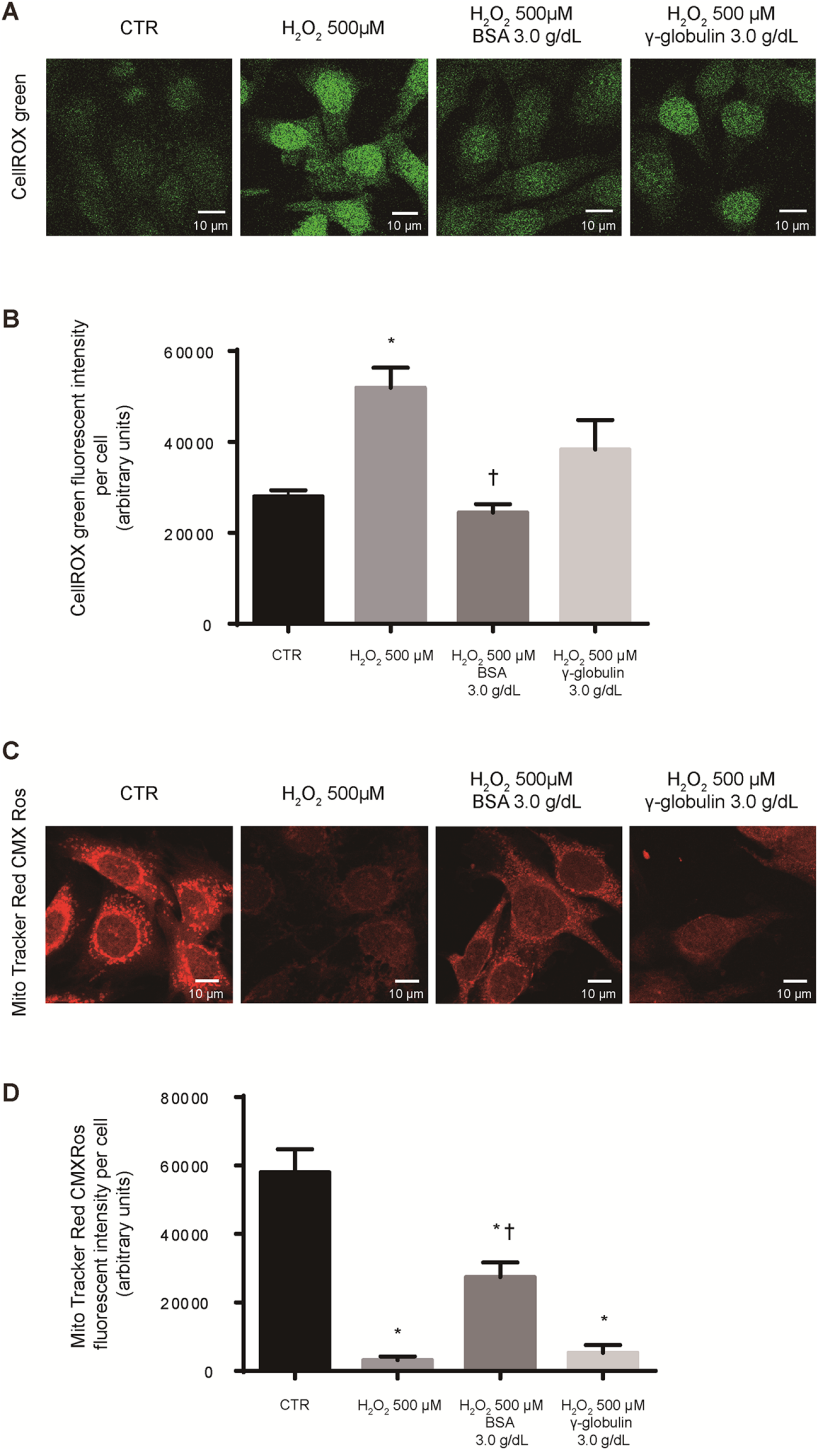
Albumin attenuated intracellular reactive oxygen species (ROS) levels and mitochondrial damage induced by hydrogen peroxide in mouse mesangial cells. (A) Representative image of intracellular ROS levels detected by CellROX green staining in SV40 MES-13 cells after 6-h exposure to hydrogen peroxide (500 μM) with phosphate buffered saline (PBS), bovine serum albumin (BSA, 3.0 g/dL) or γ-globulin (3.0 g/dL). As a control (CTR), we used cells that were not exposed to hydrogen peroxide. For quantification, fluorescent intensities per cell were measured using ImageJ software (B), (C) Representative image of membrane potential-dependent Mito Tracker Red CMXRos staining in SV40 MES-13 cells after 6-h exposure to hydrogen peroxide (500 μM) with PBS, BSA, or γ-globulin. As a control (CTR), we used cells that were not exposed to hydrogen peroxide. For quantification, fluorescent intensities per cell were measured using ImageJ software (D). Data are presented as the mean ± standard error. (n = 8 imaged fields for each condition). * *P* <0.05 vs. CTR, † *P* <0.05 vs. H_2_O_2_ 500 μM.

Second, we analyzed intracellular ROS and mitochondrial damage induced by oxidative stress using CellROX staining and Mito Tracker Red CMXRos staining, membrane potential-dependent mitochondrial marker, in mouse mesangial cells. Similar to the results obtained using DHE, we observed increased intracellular ROS and mitochondrial damage after 500 μM hydrogen peroxide stimulation, and when cells were pretreated with 3.0 g/dL BSA, they were decreased. In addition, the degree of reduction was stronger than when cells were pretreated with γ-globulin (Fig 5A–5D).

**Fig 6:**
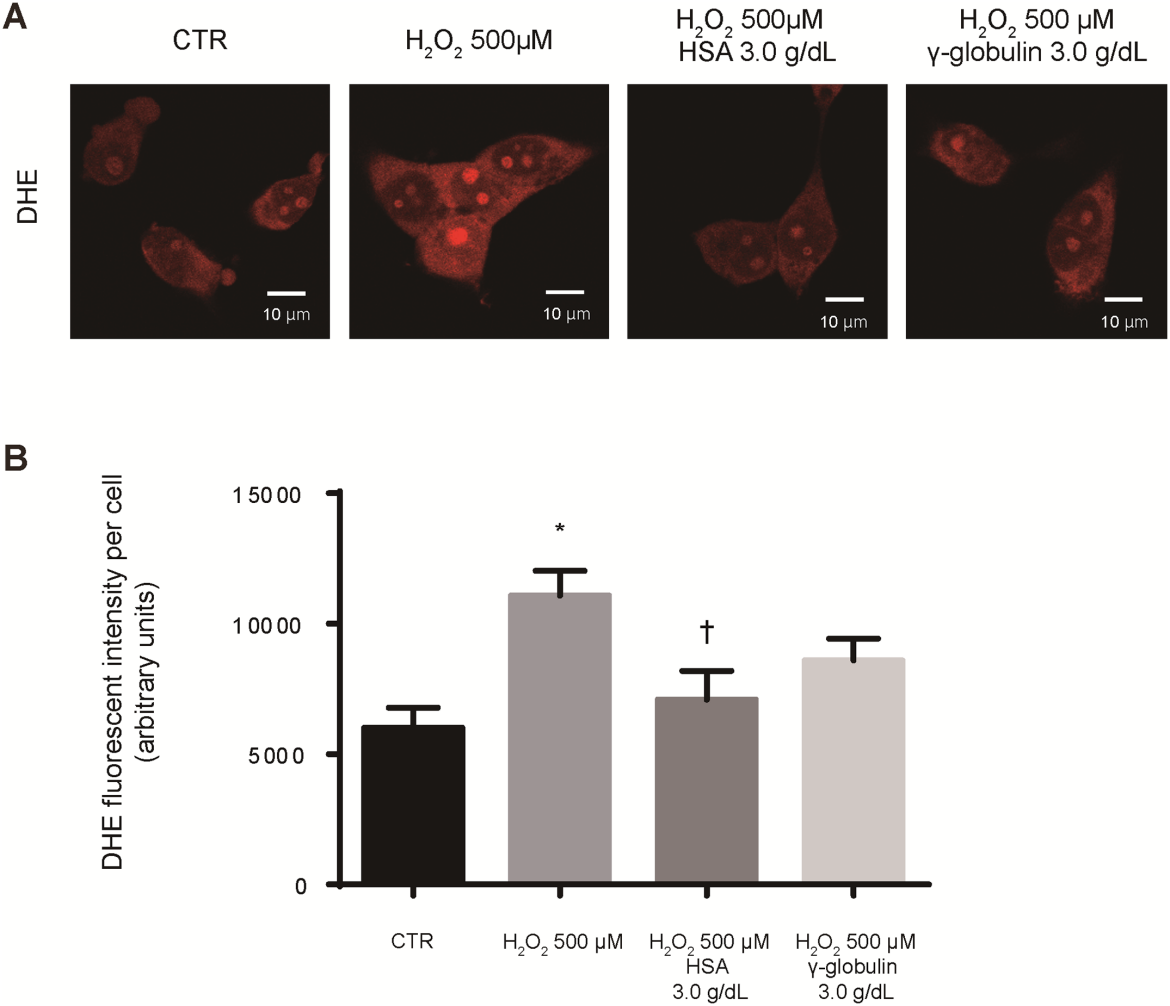
Albumin led to the reduction of intracellular superoxide anion levels induced by hydrogen peroxide in human kidney cells. (A) Representative image of intracellular superoxide anion levels as detected by dihydroethidium (DHE) staining in human embryonic kidney (HEK) 293FT cells after 6-h exposure to hydrogen peroxide (500 μM) and pretreatment with phosphate buffered saline (PBS), human serum albumin (HSA, 3.0 g/dL) or γ-globulin (3.0 g/dL). As a control (CTR), we used cells that were not exposed to hydrogen peroxide. (B) The DHE fluorescent intensity per cell was quantified. HEK 293 FT cells were exposed to 500 μM hydrogen peroxide for 6 h with PBS, HSA, or γ-globulin. As a CTR, we used cells that were not exposed to hydrogen peroxide. Fluorescent intensities per cell were measured using ImageJ software. Data are presented as the mean ± standard error. (n = 8 imaged fields for each condition). * *P* <0.05 vs. CTR, † *P* <0.05 vs. H_2_O_2_ 500 μM.

In order to investigate whether the antioxidant effect of albumin is observed in human as well as mouse kidney cells, we also tested human kidney cells (HEK 293FT cells). We analyzed intracellular superoxide anion levels in HEK 293FT cells following hydrogen peroxide exposure and pretreatment with PBS, HSA, or γ-globulin using DHE staining. In human kidney cells, DHE fluorescence was increased by hydrogen peroxide exposure was also attenuated by pretreatment with albumin as evident in mouse mesangial cells (Fig 6A and 6B).

## Discussion

We clearly revealed the relationship between low serum albumin and either the incidence of ESRD or the doubling of serum creatinine from baseline in patients with IgAN. Additionally, subgroup analysis demonstrated that such relationship was not affected by background comorbidities, such as age, sex, BMI, eGFR, urinary protein excretion, and pathological parameters (MEST-C score). Furthermore, *in vitro* experiments revealed that albumin suppressed intracellular ROS, and mitochondrial damage induced by hydrogen peroxide in a dose-dependent manner and suppressed more clearly than γ-globulin in mesangial cells, which were predominantly injured in patients with IgAN. These findings suggest that serum albumin may play an important role as an antioxidant on the mesangial cells and could be associated with decreased risk of ESRD.

The relationship between urinary protein and renal prognosis is well known, but not much is known about the relationship between serum albumin and renal prognosis. A few clinical studies have shown an association between low serum albumin and the progression of chronic kidney disease [3,19, 28–30]. In the retrospective Japanese cohort study of 2,283 patients with IgAN, patients with serum albumin <4.0 g/dL had a 1.94-fold higher risk of developing ESRD than those with serum albumin ≥4.0 g/dL [3]. It was also reported that low serum albumin was associated with a worse renal prognosis in a sub-analysis of the prospective, multinational, double-blind, randomized study in 1,513 patients with diabetic nephropathy [29]. In this prospective study, serum albumin was significantly associated with a worse renal prognosis even in the groups with less than 2 g/g urinary albumin-creatinine ratio [29]. In recent study, Lang et al. showed that lower serum albumin levels were independently associated with kidney function decline in elders between the ages of 70 and 79 years, independent of urine albumin and measured inflammatory markers [30]. Our results that low serum albumin was associated with the prognosis of IgAN were compatible with these clinical findings.

In basic research, a previous experimental study reported antioxidant properties of serum albumin. Compared with control rats, albumin-deficient rats after bile duct ligation had significantly shorter survival, and as a mechanism, in albumin-pretreated endothelial cells, intracellular ROS induced by lipopolysaccharide was reduced [31]. These findings were consistent with our study, which indicated albumin had an antioxidant property for mesangial cells and was associated with a prognosis of the patients with IgAN.

Serum albumin is the most abundant intravascular protein and the major intravascular antioxidant. It exerts antioxidant functions due to its multiple ligand-binding capacities and free radical-trapping properties mediated by a single free thiol group [12,32]. Serum albumin can bind free metal ions, and limit the production of ROS induced by free metal ions (essentially copper and iron), which are critical in accelerating the production of free radicals through the Fenton reaction [33]. Moreover, serum albumin contains one reduced cysteine residue (Cys34) that constitutes the largest pool of thiols in the circulation [32]. Oxidation of Cys34 leads to the formation of sulfenic acid (RSOH), sulfinic (RSO2H) or sulfonic acid (RSO3H) [11]. Therefore, reduced Cys34 in serum albumin has an important redox regulator role in extracellular compartments [34]. In patients with IgAN, it was reported that intravascular AOPPs, markers of oxidative stress, were high and levels of AOPPs were associated with the decline of renal function [16].

In addition, results of an *in vitro* experiment reported that mRNA encoding extracellular matrix protein was increased by the oxidative stress of hydrogen peroxide in mesangial cells [35]. These results suggest the important roles of the oxidative stress in the progression of IgAN, thus the antioxidant effect of albumin may have a protective influence on the progression of IgAN. To clarify the antioxidant activity of albumin in kidney cells, we conducted *in vitro* experiments. It has been reported that ROS in glomeruli, particularly hydrogen peroxide, increases in rats with Thy 1 nephritis, which is an IgAN model commonly used in animal experiments [36]. Therefore, we focused on cytotoxicity induced by hydrogen peroxide. We found in the present experiment that ROS produced in mesangial cells exposed to hydrogen peroxide is attenuated by extracellular albumin. Furthermore, mitochondrial damage is alleviated, and this protective effect is also similar in human kidney cells. As a possible mechanism, we thought that albumin might act as a buffer against extracellular oxidative stress produced in inflamed glomeruli, thus attenuating intracellular ROS and reducing mitochondrial damage, thereby breaking the vicious circle of increased oxidative stress in kidney cells. The antioxidant effect of albumin in mesangial cells may be one of the mechanisms of its protective effect on the progression of IgAN. In our study, we have shown that the intracellular ROS, which occur from the oxidative stress with hydrogen peroxide, are attenuated in albumin-pretreated mesangial cells, and this may be one of the mechanisms for the association between low serum albumin and the incidence of ESRD in patients with IgAN.

This study has a number of strengths. It consisted of a large number of participants and had long follow-up periods with not only the doubling of serum creatinine but also hard ESRD endpoint. Few studies have focused on the association between serum albumin by itself and prognosis of IgAN because serum albumin is usually thought of in connection with proteinuria or malnutrition or inflammation. Additionally, these previous studies were limited by the lack of adjustment for quantitative proteinuria and not using an internationally accepted pathologic grading system [3], such as the Oxford classification. Furthermore, in an *in vitro* experiment, we reported a reduction of the intracellular ROS and mitochondrial damage in albumin-pretreated mouse mesangial cells incubated with hydrogen peroxide and a similar effect was obtained in human kidney cells. These results strengthen the idea that there is an association between serum albumin and renal prognosis in patients with IgAN and the strategy to increase serum albumin without increasing protein urine might be a useful way to attenuate progression of IgAN.

This study also has several limitations that need to be considered. First, serum albumin was measured only once at baseline examination. During the follow-up, the levels could have changed because of lifestyle or medication alterations, and misclassification of serum albumin levels might occur. Therefore, the shown association could have been weakened by this misclassification. Second, although some potential confounding variables were adjusted for in the multivariable-adjusted analyses, there were still residual confounding variables at baseline, such as unmeasured factors like genetic background. Third, even though we adjusted for urinary protein excretion and histological severity, we might not completely remove their confounding effects. Finally, it should be noted that our results have potential limitations on generalizability to CKD patients other than those with IgAN. However, it has been reported that the impact of oxidative stress on renal disease progression is not a specific phenomenon in patients with IgAN but also is evident in diabetic nephropathy [37]. Thus we believe that the renoprotective effect demonstrated through the antioxidant capacity of albumin can be extrapolated to patients with other CKD spectrum as well as IgAN. Further studies are required to elucidate the association between serum albumin and the incidence of ESRD in patients with IgAN, and to clarify the pathophysiology of serum albumin in patients with IgAN.

## Conclusions

Albumin had an antioxidant effect on mesangial cells, and low serum albumin was an independent risk factor for the incidence of ESRD in patients with IgAN after adjustment for age, sex, SBP, urinary protein excretion, BMI, eGFR, total cholesterol, triglycerides, and pathological parameters. Taking together with *in vitro* experiments, the strategy to increase serum albumin may be a useful way to attenuate progression of IgAN.

## Supporting information

S1 Table Multivariable-adjusted hazard ratios for the incidence of ESRD. (DOCX)
