# Supplementary material for: Association between serum albumin level and incidence of end-stage renal disease in patients with Immunoglobulin A nephropathy: A possible role of albumin as an antioxidant agent
Source: PLoS One. 2018 May 24;13(5):e0196655. doi: 10.1371/journal.pone.0196655 (PMC5968404; doi:10.1371/journal.pone.0196655)
Supplement: S1 Table — (DOCX) [file pone.0196655.s001.docx]

**Association between Serum Albumin Level and Incidence of End-Stage Renal Disease in Patients with Immunoglobulin A Nephropathy: A Possible Role of Albumin as an Antioxidant Agent**

Yasuhiro Kawai, MD,^1^ Kosuke Masutani, MD, PhD,^1^ Kumiko Torisu, MD, PhD,^1^

Ritsuko Katafuchi, MD, PhD,^2^ Shigeru Tanaka, MD, PhD,^1,3^ Akihiro Tsuchimoto, MD, PhD,^1^ Koji Mitsuiki, MD, PhD,^4^ Kazuhiko Tsuruya, MD, PhD,^1,5 *^ Takanari Kitazono, MD, PhD^1^

1. Department of Medicine and Clinical Science, Graduate School of Medical Sciences, Kyushu University, Fukuoka, Japan

2. Kidney Unit, National Fukuoka-Higashi Medical Center, Koga, Fukuoka, Japan

3. Division of Internal Medicine, Fukuoka Dental College, Fukuoka, Japan

4. Division of Nephrology and Dialysis Center, Japanese Red Cross Fukuoka Hospital, Fukuoka, Japan

5. Department of Integrated Therapy for Chronic Kidney Disease, Graduate School of Medical Sciences, Kyushu University, Fukuoka, Japan

*** Corresponding author**

Kazuhiko Tsuruya, MD, PhD

Department of Integrated Therapy for Chronic Kidney Disease,

Graduate School of Medical Sciences, Kyushu University

3-1-1 Maidashi, Higashi-ku, Fukuoka 812-8582, Japan

Tel: +81-92-642-5843, Fax: +81-92-642-5846

E-mail: tsuruya@intmed2.med.kyushu-u.ac.jp

# Table S1. Multivariable-adjusted hazard ratios for the incidence of ESRD

|  |  |  | Age- and sex-adjusted | |  | Multivariable-adjusted (model 1)^a^ | |  | Multivariable-adjusted (model 2)^b^ | |
| --- | --- | --- | --- | --- | --- | --- | --- | --- | --- | --- |
| Variable | No. of Events | No. of Patients | HR (95% CI) | *P* value |  | HR (95% CI) | *P* value |  | HR (95% CI) | *P* value |
| Every 1.0 g/dL decrease in albumin | 71 | 658 | 4.26 (3.25–5.55) | <0.001 |  | 2.43 (1.36–4.44) | 0.003 |  | 2.06 (1.09–3.99) | 0.03 |

^a^Model 1: Adjusted for age, sex, SBP, urinary protein excretion, BMI, eGFR, total cholesterol, triglycerides, and log CRP.

^b^Model 2: Model 1 plus M, E, S, T, and C.

Abbreviations: BMI, body mass index; C, crescent score; CI, confidence interval; CRP, C-reactive protein; E, endocapillary hypercellularity score; eGFR, estimated glomerular filtration rate; ESRD, end-stage renal disease; HR, hazard ratio; M, mesangial hypercellularity score; S, segmental glomerulosclerosis score; SBP, systolic blood pressure; T, tubular atrophy/interstitial fibrosis score.
